# High coverage of mass drug administration for lymphatic filariasis in rural and non-rural settings in the Western Area, Sierra Leone

**DOI:** 10.1186/1756-3305-3-120

**Published:** 2010-12-16

**Authors:** Mary H Hodges, Samuel J Smith, Daniel Fussum, Joseph B Koroma, Abdul Conteh, Mustapha Sonnie, Santigie Sesay, Yaobi Zhang

**Affiliations:** 1 Helen Keller International, PO Box 369, Freetown, Sierra Leone; 2 District Health Management Team, Ministry of Health and Sanitation, Sierra Leone; 3 World Health Organization, P. O. BOX 529, Freetown, Sierra Leone; 4 Neglected Tropical Disease Control Program, Ministry of Health and Sanitation, Sierra Leone; 5 Helen Keller International, Regional Office for Africa, Dakar, Senegal

## Abstract

**Background:**

Lymphatic filariasis elimination programs are based upon preventative chemotherapy annually in populations with prevalence more than or equal to 1%. The goal is to treat 80% of the eligible, at risk population yearly, for at least 5 years, in order to interrupt transmission and prevent children from becoming infected. This level of coverage has been a challenge in urban settings. Assessing the coverage in a rapidly growing urban/non-rural setting with inadequate population data is also problematic. In Sierra Leone, a 5-day preventative chemotherapy campaign was carried out in the Western Area including the capital: Freetown. An intensive, social mobilization strategy combined traditional and modern communication channels. To aid dissemination of appropriate information Frequently Asked Questions were developed and widely circulated. The population of the Western Area has grown faster than projected by the 2004 National Census due to the post-war settlement of internally displaced persons. As a reliable denominator was not available, independent monitoring was adapted and performed "in process" to aid program performance and "end process" to assess final coverage.

**Results:**

In 5 days 1,104,407 eligible persons were treated. Using the projected population from the 2004 census this figure represented coverage of 116% in the Urban Western Area and 129% in the Rural Western Area. Independent monitors interviewed a total of 9,253 persons during the 2 End Process days representing 1% of the projected population. Of these, 85.8% recalled taking both ivermectin and albendazole (Urban: 85.2%, Rural: 87.1%). No serious adverse drug reactions were reported.

**Conclusion:**

The paper presents the key elements of success of the social mobilization and implementation strategy and describes the independent monitoring used to estimate final coverage in this urban/non-rural setting where the current population size is uncertain. This implementation strategy and Independent Monitoring tool could be useful in similar, rapidly growing cities implementing lymphatic filariasis elimination programs.

## Background

Preventative chemotherapy and transmission control (PCT) with albendazole and ivermectin (or diethylcarbamazine) annually in populations 'at risk' where lymphatic filariasis (LF) prevalence of ≥1% is the basis of LF elimination programs [1]. The goal is to treat at least 80% of the eligible, at risk population yearly, for at least 5 years, in order to interrupt transmission of LF by making the infectious microfilaria (MF) stages unavailable to the mosquito vectors [2]. Pregnant women, the unwell and children under five years are not eligible for treatment. This ultimately leads to elimination when MF falls to less than 1% in many communities especially in areas where *Anopheles *mosquitoes are the main vectors. In Sierra Leone, the prevalence of LF by immuno-chromatographic tests (ICT) was found to be 23% overall, and it was 7% in the Rural Western Area (RWA) and 12% Urban Western Area (UWA): the capital Freetown [3]. The UWA and RWA are politically defined administrative areas. Although the RWA has many rural villages, there are some areas with piped water and electricity. Similarly in the UWA, there are many areas still with typical rural characteristics. Therefore these areas are best described as non-rural.

The national LF elimination program evolved from the national onchocerciasis control program in 2007 by adding albendazole to the Community Directed Treatment with Ivermectin (CDTI) to become Community Directed Treatment with Ivermectin plus Albendazole (CDTI+). Normally CDTI+ is performed by community volunteers after they have taken a "village census" and takes 6-8 weeks to complete. The national LF elimination program scaled up geographical coverage to include all districts excluding the UWA in 2008.

In the RWA, CDTI+ was conducted in 2008 but poor coverage achieved: 22.7% (Kamara et al. unpublished data). This poor coverage illustrated that many parts of the RWA may no longer be "rural" due to the mass influx of internally displaced persons during the recent war 1991-2002, who have since settled there. The poor coverage also illustrated the difficulty in implementing CDTI+ in non-rural settings as reported by other countries [4-7]. Effective social mobilization and awareness of LF are known to be the key to improved coverage for LF elimination programs [8,9]. The implementation of CDTI+ in the RWA, 2008 also highlighted the difficulty in implementing CDTI+ with community volunteers (un-paid) in non-rural settings.

The National Neglected Tropical Disease (NTD) Task Force decided to change strategy in the UWA and RWA and utilize a modification of the National Immunization Day (NID) strategy for 2010 and scheduled 5 day implementation by paid community health workers in a street by street and fixed health center distribution campaign. Recent experience in Sierra Leone with multiple NID campaigns against measles, polio (4 rounds) and yellow fever have strengthened the administrative links between District Health Management Teams (DHMT), their peripheral health units (PHUs) and in turn an extensive network of community health workers (CHWs). Building on lessons learned the LF-campaign was synchronized to occur immediately following the Mother and Child Health Week so that the same peripheral health personnel, CHWs and channels of communication could be used to disseminate information and materials to peripheral health units and onwards to their catchment communities.

The objective was to administer ivermectin and albendazole to a minimum of 80% of the eligible WA population. The actual populations of the UWA and RWA are greater than the 2004 Census projections due to the large influx of internally displaced persons during and after the war. As no pre-intervention census was undertaken, independent monitoring was implemented to assess the final coverage. This paper reports on the implementation strategy, social mobilization, the high coverage achieved in the UWA and RWA, and relative cost needed for each person treated.

## Methods

### Information, education and communication (IEC)

Suitable IEC material for the urban and semi-urban setting was developed by the NTD Task Force and pre-tested. Frequently Asked Questions (FAQs) were developed to ensure that appropriate, consistent and culturally sensitive messages about LF, the NID campaign and exclusion criteria were disseminated to the health sector and wider public to minimize misinformation. The FAQs were repeatedly tested, improved, and disseminated in the 2 week pre-campaign launch responding to public feedback from community meetings, phone-ins or SMS messages following radio discussions. These FAQs clearly explained to leaders the participation expected of them to facilitate the campaign such as the provision of drinking water within their communities. As the low income and very-low income populations are known to be most at risk of *Wuchereria bancrofti *microfilaraemia [10] special emphasis was given in the FAQs requesting the more literate to inform vulnerable groups in their neighborhoods and workplaces and to explain to them how to access treatment during the campaign.

**Figure 1:**
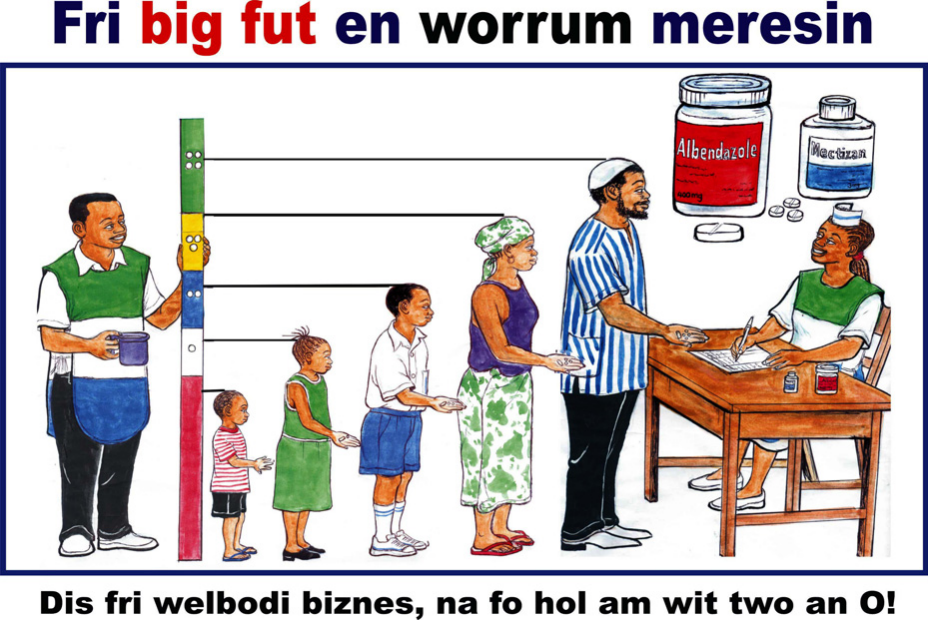


New posters of a 'PCT image' and a 'Tag' were developed and pre-tested to suit the urban context where higher literacy rates suggested that a more critical attitude towards PCT would be encountered. The 'Tag' in the local language emphasized that the medicines were free of charge and treated "worrums" as well as "bigfut", the local names for STHs and LF. The new 'PCT image' graphically explained the treatment protocol, illustrating a dose pole and family members receiving different dosages of ivermectin plus one albendazole (figure 1). The 'PCT image and Tag' was reproduced in A4 laminated cards so that all councilors, PHU staff, CHWs, zonal supervisors and independent monitors could refer to the same image consistently. This 'PCT image and Tag' was advertised in a leading newspaper for the duration of the campaign together with an editorial on the first day.

### Social mobilization

Social mobilization was intense, coordinated and comprehensive over a 3-week period. A full-day advocacy meeting was held by the District Health Management Team-Western Area (DHMT-WA) which explained the justification for the LF campaign, the disease, implementation strategy, exclusion criteria, target priorities and time-line in order to solicit the full cooperation of senior leaders. All community radio stations were supplied copies of an interview based upon the FAQs and additional radio discussions, jingles and spot messages for broadcasting the week prior and during the campaign. This enabled an interactive platform with the public via phone-ins and SMS messages to address misunderstandings regarding "bigfut". The public's most frequent concern related to traditional beliefs and witchcraft. The Director of Education, Western Area notified all schools of this campaign in advance allowing head teachers time to inform parents and obtain consent for the CHWs to enter schools and treat children. The key elements of social mobilization are shown in Appendix 1.

### Reporting

Reporting forms had been simplified from the CDTI+ returns. Tally sheets were developed that required only the recipient's sex, the number of ivermectin tablets administered and the total of ivemectin and albendazole dispensed by that CHW team on a single day. The distribution of CHWs per PHU was based upon the size of the PHU catchment area and previous NID experience of the 'Hard to Reach' areas. Each CHW team was estimated to need to treat approximately 218 persons daily to reach all eligible persons. A total of 1,030,000 doses was initially allocated prior to the launch. Summary sheets were made in each of the 120 PHUs, compiled by the zonal supervisors for daily de-briefing with the DHMT-WA and independent monitors at the end of the day.

### Training

Training of health service supervisors following the advocacy meeting enabled each of the 120 health units in the WA to hold a community meeting in participation with civil, traditional and religious leaders in their catchment areas. At these meetings the FAQs were used to guide discussion and provide satisfactory answers to often challenging questions. Training by the PHU supervisors of 1,700 Community Health Workers (CHWs) was performed the day before the commencement of the PCT campaign. This rapid training-implementation strategy had been found successful in the previous schsitosomiasis campaign in June 2009 (Hodges et al. unpublished data). Strict adherence to a training agenda using the LF Training Manual and FAQs were followed by practice using dose poles and tally sheets to ensure consistency of best practice throughout the WA.

### Implementation

Implementation was performed by CHWs working in teams of two (a total of 850 teams): one to recruit, organize and measure the height of participants and the other to record and dispense drugs by directly observed treatment. A launching ceremony filmed by National Television crews and broadcast that evening targeted the "middle classes" who witnessed senior medical and political figures participating in "directly observed treatment". The Thursday launch enabled CHWs to access all schools and colleges before the weekend, the children then sensitized their parents further when they returned home from school. Prisons, army, council workers (street cleaners) and police barracks were early institutions reached on days 1-2. Street by street coverage was performed from day 1. The mosques were attended by CHWs after prayers on Friday (day 2) and churches on Sunday (day 4). By Monday (day 5) the more 'sophisticated" commercial areas were targeted together with "mopping up" of areas less well covered that had been identified by personal contacts, monitors or supervisors. The key elements of implementation are shown in Appendix 2.

Although the initial distribution of drugs was in excess of that calculated to reach 80% of eligible population projections (1,030,000 versus 743,025) by campaign day 2 it was evident that more drugs would be required to meet the need. Additional supplies were sourced by the NTDCP from the provinces and by a donation from a local NGO that enabled health units to be re-stocked during Day 3 enabling PCT to continue for Days 4 and 5.

### Independent Monitoring

The existing population data based upon normal growth from the National Census 2004 was not considered a reliable denominator to assess final coverage. Community Drug Distributors have been used to carry out census in urban settings prior to commencement of CDTI in other countries, however, this was not considered feasible in the UWA and RWA due to the size and transient nature of the populations in many of these new post-conflict settlements. Therefore, independent monitoring was used to evaluate the coverage.

Independent monitoring developed for the recent polio campaigns by the WHO was adapted for the LF campaign using a cluster sampling method stratified by district. The primary sample clusters were the enumeration areas (EAs): collections of households (defined as a group of people who eat from the same cooking pot) grouped within defined administrative boundaries from the 2004 census. The list of EAs was used for the selection of the 30 clusters using probability proportional to size (PPS) for RWA and for UWA. The clusters were physically located by the independent monitors.

Twenty independent monitors were trained for one day and worked from days 3-5 of the PCT campaign with in-process monitoring and two days of end-process monitoring. Five communal "locations" in each site were sampled representing places such as bus stops, local markets, school environs, churches or mosques, petrol stations, shops, farms, workshops, entertainments centers or street water pumps. A minimum of 30 and a maximum of 50 people were interviewed in each location depending upon the size of the location and asked whether they had taken ivermectin and albendazole prompted by the 'PCT image and Tag' if needed.

In addition the monitors walked 10 and 20 minutes away from the nearest health center in UWA and RWA respectively to reach the periphery of that catchment area without entering the next catchment area where they sampled a minimum of 30 and a maximum of 50 households. The first household was selected from a house representative of the locality; the second household was the one that was immediately on right of the previous household and so on. In each household the monitors distributed numbered ballot cards to "eligible" persons. A number was then randomly selected and the person with that number on the card was interviewed and the recall of that single respondent was recorded. If only one person in the house and is eligible then that person is interviewed.

On the last day of "In Process" monitoring and the Day 2 of "End Process" monitoring 30 "Hard to Reach" areas were monitored in UWA and also in RWA.

### Statistical Analysis

The data from the independent monitoring was analyzed by Chi-squared (χ^2^) test to determine significant difference of final coverage results between RWA, UWA, Day 1 'End process' and Day 2 'End process, Hard to Reach', Household or communal location at 5% level of confidence.

### Cost analysis

The costs of the PCT in the WA were analysed, and included those for social mobilization, production of IEC materials, radio broadcasts, press releases, newspaper advertisements, launching ceremony, training and allowances for CHWs and independent monitors, and supervisions by program teams. A simple cost per person treated was calculated using these costs and the total number of persons treated. The costs did not include all running costs for the Ministry of Health and Sanitation program staff and DHMT staff, such as salaries, benefits, office and vehicle expenditures. The costs for drugs were also not included in the calculation as both ivermectin and albendazole are donated by Merck and GlaxoSmithKline respectively.

## Results

Using the projected data from the national census [11] the total population of the WA is 1,187,460 (UWA: 945,501, RWA: 241,959) and the eligible population (excluding pregnant women, children under 5 years and the unwell) was estimated as WA 928,781 (UWA: 740,328, RWA: 188,453) as shown in Table 1.

**Table 1:** Reported ivermectin and albendazole treatments by area, day and cumulative coverage (%) by projected eligible population (National Populations Census 2004)

	Projected Pop	Projected Eligible Pop	Day 1		Day 2		Day 3		Day 4		Day 5		Day 1-5
			
Area			Treated	%	Treated	%	Treated	%	Treated	%	Treated	%	Treated
UWA	945,501	740,327	341,194	46	253,401	80	134,555	98	64,896	107	66,978	116	861,024

RWA	241,959	188,454	86,653	46	63,836	80	40,521	101	27,047	116	25,326	129	243,383

Total	1,187,460	928,781	427,847	46	317,237	80	175,076	99	91,943	109	92,304	119	1,104,407

### Results based upon the Population Census and DHMT records of treatments performed

The results based upon the projected eligible population from the 2004 census and the daily compilation of tallies is shown in Table 1. By Day 1, 46% of the eligible population had received treatment and by Day 2: 80%, Day 3: 99%, Day 4: 109% and Day 5: 119% coverage had been achieved. The total tally of persons treated over the 5 day campaign was 1,104,407 (UWA: 861,024, RWA: 243,383). Using these records 116% of the eligible population had been treated in the UWA and 129% in the RWA.

### Results based upon the Independent Monitoring

The "in process" independent monitoring started on campaign day 3 and showed that 72.5% interviewees (3268/4510) on Day 3, 79.5% (3674/4622) on Day 4 and 84.9% (3398/4003) on Day 5 recalled taking both ivermectin and albendazole. The results of the 2 days of end process independent monitoring are summarized in Table 2. The sample size of those interviewed represented 1.00% of the eligible target population (UWA: 0.86%, RWA: 1.55%) and represented 0.84% of the persons treated (UWA: 0.74%, RWA: 1.20%).

**Table 2:** Number of persons interviewed by Household or by Location and recalling taking both ivermectin and albendazole by area, day and compliance (%) for Day 1 and Day 2 End Process.

	Sample size as % of treatments	Sample size as % of eligible pop	Day 1: End Process			Day 2 End Process: Hard to Reach			Total Day 1 & 2: End Process		
**UWA**			**Interview**	**Recall**	**%**	**Interview**	**Recall**	**%**	**Interview**	**Recall**	**%**

Household			460	371	80.7	530	418	78.9	990	789	79.7

Location			2734	2332	85.3	2613	2275	87.1	5347	4607	86.2

Subtotal	0.74%	0.86%	3194	2703	84.6	3143	2693	85.7	6337	5396	85.2

**RWA**											

Household			267	236	88.4	281	244	86.8	548	480	87.6

Location			1056	907	85.9	1308	1150	87.9	2364	2057	87.0

Subtotal	1.20%	1.55%	1323	1143	86.4	1589	1394	87.7	2912	2537	87.1

Total	0.84%	1.00%	4517	3846	85.1	4732	4087	86.4	9249	7933	85.8

A total of 9249 eligible persons (UWA: 6337, RWA: 2912), were interviewed in the 2 combined "End Process" days. Of these 7933 persons recalled taking both ivermectin and albendazole (UWA: 5396, RWA: 2537) equivalent to 85.9% final coverage (Urban WA: 85.2%, Rural WA: 87.1%). There was a significant difference in coverage amongst persons interviewed in households in UWA versus both those interviewed on location at End process in UWA and versus those interviewed on location at End process in RWA (p < 0.005). There was no significant difference in coverage between those interviewed at home versus location in the RWA (p = 0.72). There was no significant difference in coverage amongst persons interviewed at End Process Day 1 versus Day 2: Hard to Reach Areas in the UWA (p = 0.49) or the RWA (p = 0.58). There was a significant difference in overall coverage at End process in UWA versus RWA (p < 0.05).

### Severe Adverse Reactions

No serious adverse events were formally reported to the Pharmacy Board by the PHU staff. One person with severe vomiting on Tasso Island had required oral rehydration and then intravenous rehydration was informally reported. There were 146 minor drug reaction reporting 238 symptoms (males 126: females: 110) mostly itching: 79, swelling: 59, rashes: 25, headache: 16, dizziness: 13, abdominal discomfort: 10 and vomiting: 9 lasting less than 3 days. Reports of polyuria (6), erectile dysfunction (3) and numbness of the limbs (1) were made not previously reported in the literature.

### Cost

This NID campaign cost $132,000 including the independent monitoring but excluding standing costs for the NTDCP, DHMT-WA and drugs. This represents $0.12 per person treated.

## Discussion

The challenge of obtaining high coverage in urban settings has been reported in Orissa: 42% [4], Haiti: 68.3% [5], Columbo: 53.4% [6] and Tamil Nadu 73% [7]. Using the 2004 census projections, PCT coverage in the UWA would have been reported as 116%, while using independent monitoring data, PCT coverage was 85.2%. Likewise the RWA would have been reported as 129% based upon census projections while it was 87.1% by independent monitoring. The disparity is a reflection of the population growth in the WA since 2004 due to the post war settlement, especially so for the RWA which is now a rapidly growing non-rural setting in many parts. The coverage was remarkably higher compared with 22.7% in the previous CDTI+ performed in the RWA, 2008 (Kamara et al. unpublished data) and with 72.9% in the other 12 Districts of Sierra Leone achieved in the last round of CDTI+, 2009.

The remarkable improvement in coverage from 22.7% with CDTI+ in 2008 to 87.1% with this NID strategy in the RWA may also be a reflection of increased human resource capacity within the health sector following the Government of Sierra Leone launch of Free Health care in 2010 and considerable investments from other donors since the end of the war. In addition the recent Yellow fever campaign in December 2009 and 4 polio campaigns March-May 2010 had strengthened the DHMT-WA their administrative structures, data sources and communication links with their PHU staff and CHWs making them "campaign ready". Motivating CHWs with a small daily remuneration over a short time-frame was also highly appreciated and improved their performance.

Although there was a significantly lower overall coverage of those interviewed in household in the UWA versus those interviewed in locations in the UWA and in locations in the RWA the final coverage was still as high as 79.5%. There was no significant difference in coverage amongst those interviewed on the 'End process' Day 1 and Day 2 in the 'Hard to Reach' areas although the lowest coverage was 78.9% in households in the 'Hard to reach", UWA.

The more "modern" approaches to community sensitization such as the recurrent distribution of FAQs by community radios and internet reached individuals and institutions throughout Sierra Leone that had been unaware of CDTI+ through more "traditional" methods. There might have been some internal migration into the WA in order to receive PCT during these 5 days because of the high visibility of this campaign but these individuals would probably not have been represented in the 2 days End Process monitoring as they would have returned to their districts.

The interactive use of community radio with follow-up discussions based upon phone-in questions and SMS messages together with the extension, intensive social mobilization by all PHU staff in collaboration with traditional, religious and council leaders enabled feedback to the NTDCP and rapid revision of the FAQs. This led to a much better understanding of the current concerns and beliefs of the urban/non-rural population and potential challenges during the campaign in time for these to be addressed during the training of the CHWs the day before PCT implementation commenced. The hand-held PCT image and Tag facilitated the discussion by the CHWs within the communities during implementation.

The pharmacovigilance teams received no reports of serious adverse effects from the PHU staff during the campaign although polyuria, erectile dysfunction and numbness of the limbs were reported. Despite achieving such high coverage there are still lessons to be learnt. Most notable was the perception amongst the young adults that ivermectin and albendazole might have an adverse effect upon fertility or potency. In addition the influx of individuals into non-rural setting for treatment during a highly visible campaign could be anticipated even where PCT has been implemented in the surrounding provinces and a reserve of drug stocks made available to avoid loss of momentum. Cinemas, tele-centers and sports clubs could be included as distribution points in future campaigns. Some household members waited at home for treatment even when they were aware of the CHWs distribution in their street locations. The explanation that the distribution of drugs for the LF campaign is not strictly house to house but street by street will require further attention in the FAQs in future campaigns.

The calculation of the eligible population was based upon the current estimates of 18% of the total population being under 5 years of age and 4% being either pregnant or unwell. As 1,104,407 doses were required to reach 85.8% of the eligible population of the WA the total population in the WA can be estimated as approximately 1,650,790. Since the 2004 census projected population for the WA in 2010 was 1,186,183 this would represent an additional 464,607 persons and a growth of 39% above projections.

The cost of this LF campaign at 0.12 USD per person achieving 85.2% in the UWA including social mobilization and implementation with paid community health workers compares favorably with COMBI (++) used in Tamil Nadu that achieved 73% coverage [9].

Whilst the weaknesses of the method of Independent Monitoring are well recognized by the authors it was affordable, quick, and simple and provided valuable information: final WA coverage estimate of 85.8% which would not have been obtainable from the normal reporting procedures based upon the projected population data.

## Conclusion

This NID campaign for PCT-LF with its short time frame and high visibility could be utilized by other countries implementing LF control in urban settings. The high coverage was achieved by coordinated, intense, focused social mobilization using traditional and modern strategies. The use of Frequently Asked Questions adapted and modified in the local context before and during the campaign ensured that consistent "best practice" was being followed by all involved. The 'PCT image and Tag' adapted for the urban context minimized the problems of communication between CHWs and a public unfamiliar with dose poles, protocols and ivermectin. Independent Monitoring enabled an end process coverage figure to be reported and is recommended in similar, urban/semi-urban settings for LF elimination programs where the population is rapidly growing and accurate census projections unavailable.

## Abbreviations

CDTI: Community Directed Treatment with Ivermectin; CDTI+: Community Directed Treatment with Ivermectin plus Albendazole; CHWS: Community Health Workers; DHMT: District Health Management Teams; FAQ: Frequently Asked Question; HTR: Hard to Reach; ICT: Immuno-chromatographic tests; LF: Lymphatic filariasis; NTDS: Neglected Tropical Diseases; NTDCP: Neglected Tropical Disease Control Program; NGO: Non-Governmental Organization; NIDS: National Immunization Days; M&CHW: Mother and Child Health Week; PCT: Preventative chemotherapy; PHU: Peripheral health units; RWA: Rural Western Area; UWA: Urban Western Area; USAID: United States Agency for International Development.

## Appendix 1: Key points to successful Social Mobilization: Adapted IEC materials

Posters with 'PCT Image and Tag' to explain dosage, diseases and free of charge protocol

Revised 'PCT Image and Tag' carried by all CHWs to help explain dosage protocol

Advocacy meetings for senior leaders lead by the District Health Management Team-WA including

Councilors from Freetown City Council, Western Area Rural District Council, civil society, Interreligious Council, tribal leadership council

Letters to Parliamentarians, Medical and Nursing profession, Private medical practitioners, Labor Unions and Director of Education-WA

Community meetings held by each Peripheral Health Unit following the 'FAQs script' for consistency of information

Community Radio broadcasting of discussions following the 'FAQs script' in the 2 weeks prior to the campaign

Internet circulation of repeatedly revised FAQs by email and finally Facebook to all contacts within the WA not just the health sector

Presentation of plans to all NGOs in two monthly meetings leading up to and including the launch day

Newspaper articles and advertisement with 'PCT Image and Tag'

Press Conferences at Ministry of Information and Communication attended by SL national journalists association

National Television broadcasts of the Campaign launch and progress during the campaign

## Appendix 2: Key point to successful implementation

Information sharing, early discussions and collaboration by the DHMT-WA with other stakeholders

Strong Management, planning, commitment and co-ordination by the DHMT

Capacity within the Health Sector due to increased personnel and training

Technical support from Helen Keller International Country Office developing IEC materials

Structured cascade Training of PHU and CHWs following the FAQs and LF Training Manual

Recent revised pay structure within the health sector and increased number of peripheral unit staff

Experienced National and Zonal Supervisors

Adequate and timely funding from USAID through RTI

Synchronization with the Mother and Child Health Week to assist communication and supplies

Motivation of the Health Sector: payment of incentives by the DHMT upon receipt of reports

Campaign experience following the recent Yellow Fever and 4 rounds of Polio campaigns

Daily 5 pm de-briefing at the DHMT attended by Zonal Supervisors and Independent Monitors

Good cooperation between the DHMT, National NTD Control Program and a local NGO to avert stock outs

## Competing interests

The authors declare that they have no competing interests.

## Authors' contributions

SS, JBK, AC, MS, MH and SJS were responsible for national macro-planning and implementation. SJS was responsible for micro planning and implementation at district level. SJS and AC were responsible for the data collected. MH and DF planned the independent monitoring. MH and YZ researched and prepared the manuscript and data analysis. All authors read and approved the final manuscript.
